# Identifying the best regimen for primary eradication of Helicobacter pylori: analysis of 240 cases

**DOI:** 10.1002/mbo3.1223

**Published:** 2021-10-07

Yao Chen, Qingyi Liu, Fulian Hu and Jizheng Ma. *MicrobiologyOpen*. 2020;9: e1120.

The co‐author Jizheng Ma’s affiliation was published as Department of Gastroenterology, Xiyuan Hospital, Beijing, China.

The correct details are Department of Gastroenterology, Guang’anmen Hospital, China Academy of Chinese Medical Sciences, Beijing, China.

The author apologizes for this mistake.

